# Supplementary material for: γ-BaFe2O4: a fresh playground for room temperature multiferroicity
Source: Nat Commun. 2022 Dec 27;13:7968. doi: 10.1038/s41467-022-35669-5 (PMC9794801; doi:10.1038/s41467-022-35669-5)

Supporting Information

**γ-BaFe_2_O_4_: a fresh playground for room temperature multiferroicity**

Fabio Orlandi,* Davide Delmonte, Gianluca Calestani, Enrico Cavalli, Edmondo Gilioli, Vladimir V. Shvartsman, Patrizio Graziosi, Stefano Rampino, Giulia Spaggiari, Chao Liu, Wei Ren, Silvia Picozzi, Massimo Solzi, Michele Casappa, Francesco Mezzadri*

SINGLE CRYSTAL CHARACTERIZATION

FIGURE S1: Reciprocal space of the BaFe_2_O_4_ crystal used for structural analysis. Red, green and yellow reflections belong to the three distinct twin variants, while grey spots are common to all of them. The matrix related to the present twin law is [0.5 0.5 0; 1.5 -0.5 0; 0 0 -1].


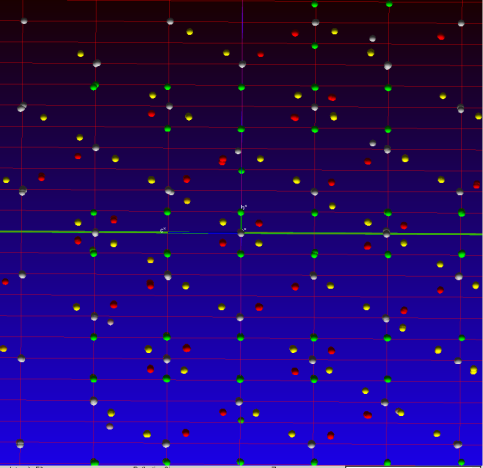


TABLE S1: Single crystal X-rays data collection, refinement and structural parameters.

| BaFe_2_O_4_, 293K  s.g. *Cmc2_1_* (36)  a = 8.437(5), b = 18.998(12), c = 5.384(3) Å  V = 863.0(9) Å^3^  ρ= 4.819 g/cm^3^  Z=8  Formula weight, 313.04 g/mol | | | | | | | | | | | | | |
| --- | --- | --- | --- | --- | --- | --- | --- | --- | --- | --- | --- | --- | --- |
| Wavelength | | | MoK_α_ (0.71073  *Å*) | | | | Reflections | | | | 1025 | | |
| μ | | | 15.537 | | | | Reflections (I > 2\s(I)) | | | | 828 | | |
| F(000) | | | 1120.0 | | | | Parameters/restraints | | | | 72/31 | | |
| h | | | 12 | | | | R1 | | | | 0.0618 | | |
| k | | | 27 | | | | R (I>2\s(I)) | | | | 0.0409 | | |
| l | | | 7 | | | | wR2 | | | | 0.0918 | | |
| θmin- θmax | | | 2.144- 31.442 | | | | wR (I > 2\s(I)) | | | | 0.0847 | | |
| Tmin-Tmax | | | 0.339-0.7462 | | | | GooF (S) | | | | 1.072 | | |
| Atom | x | y | | z | U_11_ | U_22_ | | U_33_ | U_23_ | U_13_ | | U_12_ | U_eq_ |
| Ba1 | 0 | 0.13088(8) | | -0.0002(2) | 0.0091(4) | 0.0144(6) | | 0.0245(12) | 0.0007(7) | 0 | | 0 | 0.0160(4) |
| Ba2 | 0.5 | 0.11813(7) | | 0.9643(3) | 0.0101(4) | 0.0148(6) | | 0.0207(10) | 0.0002(8) | 0 | | 0 | 0.0152(3) |
| Fe1 | 0.27980(18) | 0.04368(13) | | 0.4703(5) | 0.0097(8) | 0.0116(10) | | 0.0160(13) | 0.0004(12) | 0.0013(14) | | -0.0008(9) | 0.0124(4) |
| Fe2 | 0.29090(19) | 0.20941(11) | | 0.4889(5) | 0.0090(8) | 0.0116(10) | | 0.0152(13) | -0.0008(11) | 0.0027(15) | | 0.0010(9) | 0.0119(5) |
| O1 | 0.5 | 0.0467(7) | | 0.479(3) | 0.016(4) | 0.019(5) | | 0.020(5) | 0.001(5) | 0 | | 0 | 0.019(3) |
| O2 | 0.2091(17) | 0.1267(5) | | 0.621(3) | 0.024(4) | 0.010(4) | | 0.024(5) | 0.001(3) | 0.003(4) | | -0.002(4) | 0.019(4) |
| O3 | 0.215(12) | 0.2916(5) | | 0.647(2) | 0.012(4) | 0.016(4) | | 0.013(4) | -0.003(3) | -0.004(4) | | 0.006(4) | 0.014(2) |
| O4 | 0.5 | 0.2165(8) | | 0.591(3) | 0.023(5) | 0.024(5) | | 0.028(6) | -0.005(4) | 0 | | 0 | 0.025(4) |
| O5 | 0.2216(12) | 0.0387(6) | | 0.144(2) | 0.022(4) | 0.018(4) | | 0.014(4) | -0.003(4) | -0.002(4) | | 0.007(4) | 0.018(2) |

s.o.f.=1 for all atoms.

TEMPERATURE DEPENDENT NEUTRON POWDER DIFFRACTION

FIGURE S2: Contour plot of the neutron data collected on WISH on the back scattering detector bank with average 2θ=152.7 degrees. The data shows the development of extra reflection below T_N_=890 K.


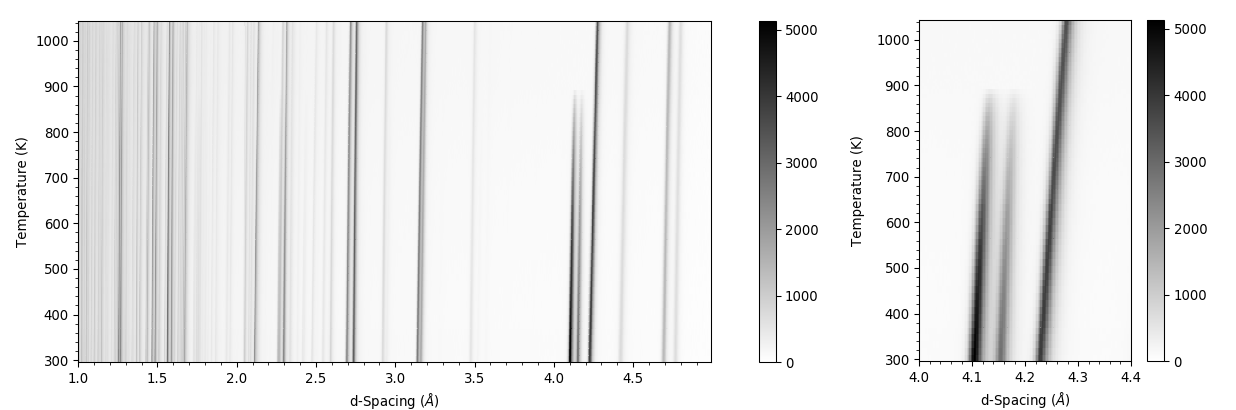


FIGURE S3: Rietveld plots of the WISH time of fight data collected on the detector banks with average 2θ of 152.7, 121.66, 90 and 58.33º at 1038 K. Observed (black crosses), calculated (red line) and difference (blue line) data are reported. The black tick marks indicate the position of the BaFe_2_O_4_ Bragg reflections. The overall reliability factors are R_p_=3.07% and _w_R_p_=3.66%.


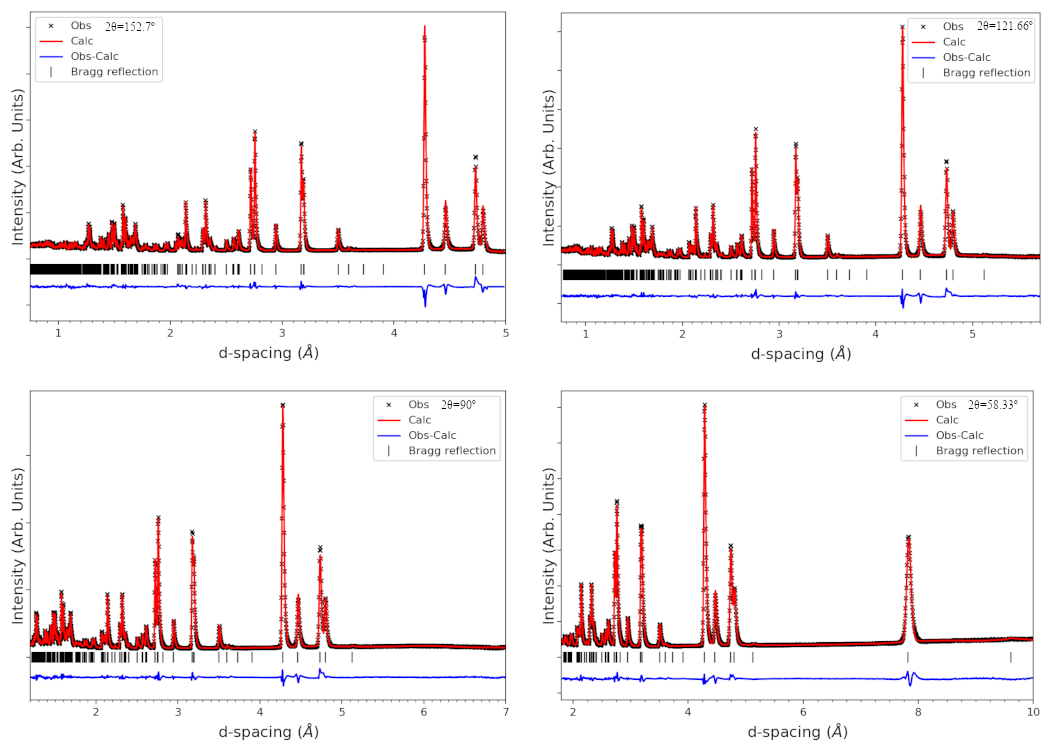


TABLE S2: Crystal and structural parameters for the refinement at 1038 K against the WISH time of fight data collected with average 2θ of 152.7, 121.66, 90 and 58.33º at 1038 K.

| BaFe_2_O_4_, 1038 K  s.g. *Cmc2_1_1'* (36)  a = 8.55488(5) Å, b = 19.19690(13) Å, c = 5.43956(3) Å  V = 893.324(10) Å^3^  ρ= 4.655 g/cm^3^  Z=8 | | | | | |
| --- | --- | --- | --- | --- | --- |
| Rp | | | 0.0307 | | |
| wRp | | | 0.0366 | | |
| GooF | | | 5.96 | | |
| Atom | x | y | | z | U_eq_ |
| Ba1 | 0 | 0.1275(4) | | 0.0164(13) | 0.0393(13) |
| Ba2 | 0.5 | 0.1194(3) | | 0.9998(14) | 0.0244(12) |
| Fe1 | 0.2830(2) | 0.04356(10) | | 0.4806(10) | 0.0350(8) |
| Fe2 | 0.28988(18) | 0.20976(9) | | 0.4846(10) | 0.0163(5) |
| O1 | 0.5 | 0.04817(16) | | 0.4540(14) | 0.070(2) |
| O2 | 0.2153(4) | 0.1278(2) | | 0.6127(10) | 0.0495(9) |
| O3 | 0.2106(4) | 0.28917(19) | | 0.6454(12) | 0.0635(11) |
| O4 | 0.5 | 0.21537(16) | | 0.5649(13) | 0.0563(19) |
| O5 | 0.2229(3) | 0.03683(17) | | 0.1492(11) | 0.0192(8) |

FIGURE S4: Rietveld plots of the WISH time of fight data collected on the detector banks with average 2θ of 152.7,121.66, 90 and 58.33º at 300 K. Observed (black crosses), calculated (red line) and difference (blue line) data are reported. The black tick marks indicate the position of the BaFe_2_O_4_ Bragg reflections. The overall reliability factors are R_p_=3.25% and _w_R_p_=4.11%.


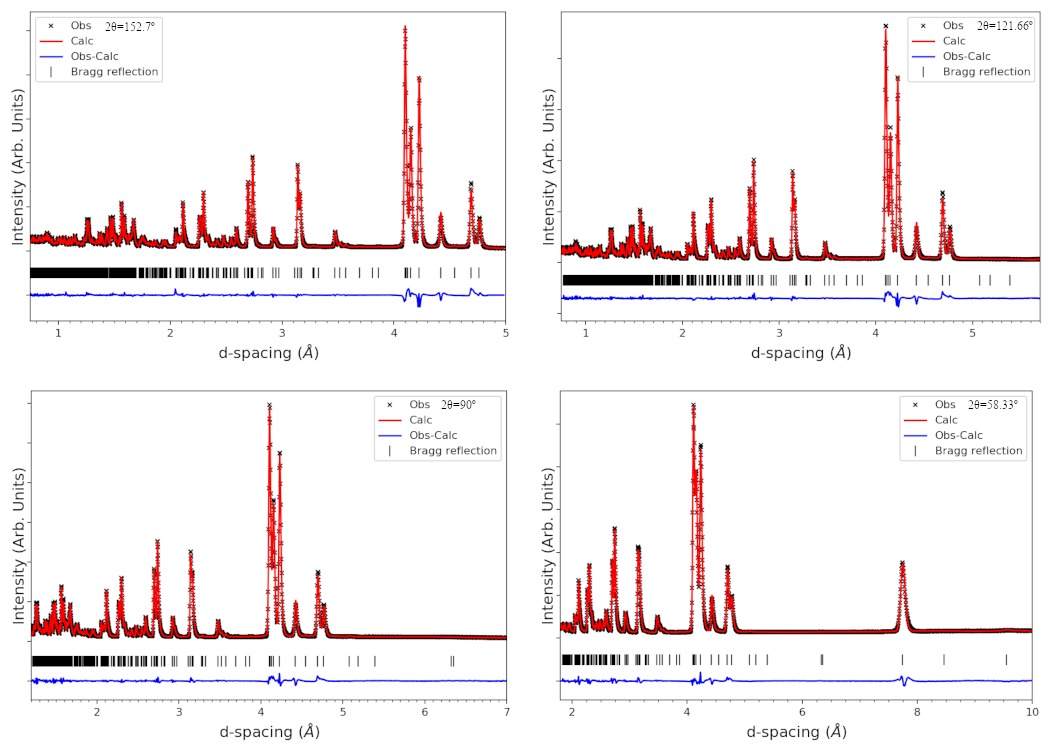


TABLE S3: Crystal and structural parameters for the refinement at 300 K against the WISH time of fight data collected with average 2θ of 152.7, 121.66, 90 and 58.33º at 1038 K.

| BaFe_2_O_4_, 300 K  s.g. *P_C_ca2_1_* (36)  a = 19.06414(12) Å, b = 8.45514(5) Å, c = 5.39014(3) Å  V = 868.837(9) Å^3^  ρ= 4.7862 g/cm^3^  Z=8 | | | | | |
| --- | --- | --- | --- | --- | --- |
| Rp | | | 0.0325 | | |
| wRp | | | 0.0411 | | |
| GooF | | | 6.72 | | |
| Atom | x | y | | z | U_eq_ |
| Ba1 | 0.3821(3) | 0.25 | | 0.000(3) | 0.0158(9) |
| Ba2 | 0.8688(3) | 0.25 | | 0.024(3) | 0.0129(9) |
| Fe1 | 0.79356(9) | 0.02985(17) | | 0.528(2) | 0.0193(6) |
| Fe2 | -0.04074(9) | 0.03894(15) | | 0.510(2) | 0.0076(5) |
| O1 | 0.79853(13) | 0.25 | | 0.520(3) | 0.0310(12) |
| O2 | 0.8770(2) | -0.0392(3) | | 0.382(2) | 0.0247(7) |
| O3 | 0.04039(17) | -0.0399(3) | | 0.355(2) | 0.0275(8) |
| O4 | -0.03521(15) | 0.25 | | 0.416(3) | 0.0316(12) |
| O5 | 0.78818(16) | -0.0275(2) | | 0.862(2) | 0.0042(7) |
| Magnetic moments | | | | | |
| Atom | Mx | My | | Mz | M_tot_ |
| Fe1 | 0 | -3.740(4)μ_B_ | | 0 | -3.740(4)μ_B_ |
| Fe2 | 0 | 3.740(4)μ_B_ | | 0 | 3.740(4)μ_B_ |

MAGNETIC CHARACTERIZATION

FIGURE S5: a-b) Zero field cooled (ZFC) and Field Cooled Cooling (FCC) measurement performed on the same sample shown in the main text with an applied field of 0.01 T and 0.1 T. c) Magnetization versus field performed on a different sample containing less than 3% wt of barium hexaferrite, as determined by XRD, showing the strong increase and sample dependence of the soft ferromagnetic component at room temperature.


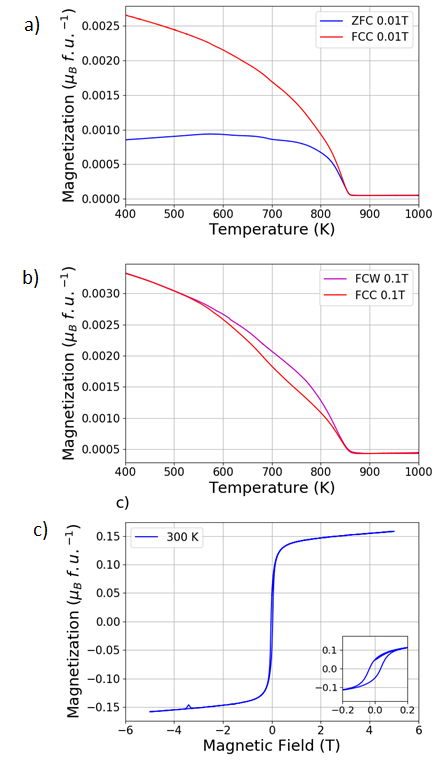


FIGURE S6: Hysteresis loops performed on a high purity BaFe_2_O_4_ sample above 800 K. The still present a weak ferromagnetic component (less than 0.01 uB f.u.^-1^) points towards the presence of Fe-rich impurities below the 0.1% wt.


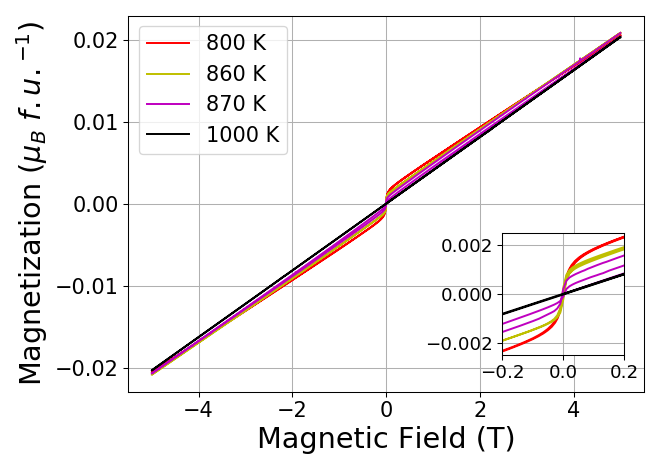


CHARACTERIZATION OF THE THIN FILM:

The sample was grown over a (110) silicon wafer covered by few nanometers of thermal oxide. X-rays diffraction points out that the film displays fiber like texture with partial [021] preferential orientation. However, has shown in Figure S17, due to marked structural similarities between the γ and β’ polymorphs, in presence of strong preferential orientation powder diffraction is not able to unambiguously allow the assignment of the phase. As a consequence, the Raman spectra of polycrystalline γ and β’ samples were collected and compared with the thin film measurement (Figure S8).

On the other hand, EDX returns atomic percentages of Ba 34 ±0.5%, Fe 66 ±0.5%, confirming the Ba/Fe ratio included between 0.50 and 0.52, in accordance with the expected value of 0.5.

Consequently, based on the present PXRD, Raman and EDX data, the thin film used for magnetic measurements appears to be clearly composed by single phase crystalline γ-BaFe_2_O_4_.

FIGURE S7: PXRD pattern of the thin film sample used for magnetic characterization (red). The computed pattern is shown in black at the bottom, where intensities were modified by the application of the March-Dollase model, allowing to account for the preferential orientation displayed by the sample.


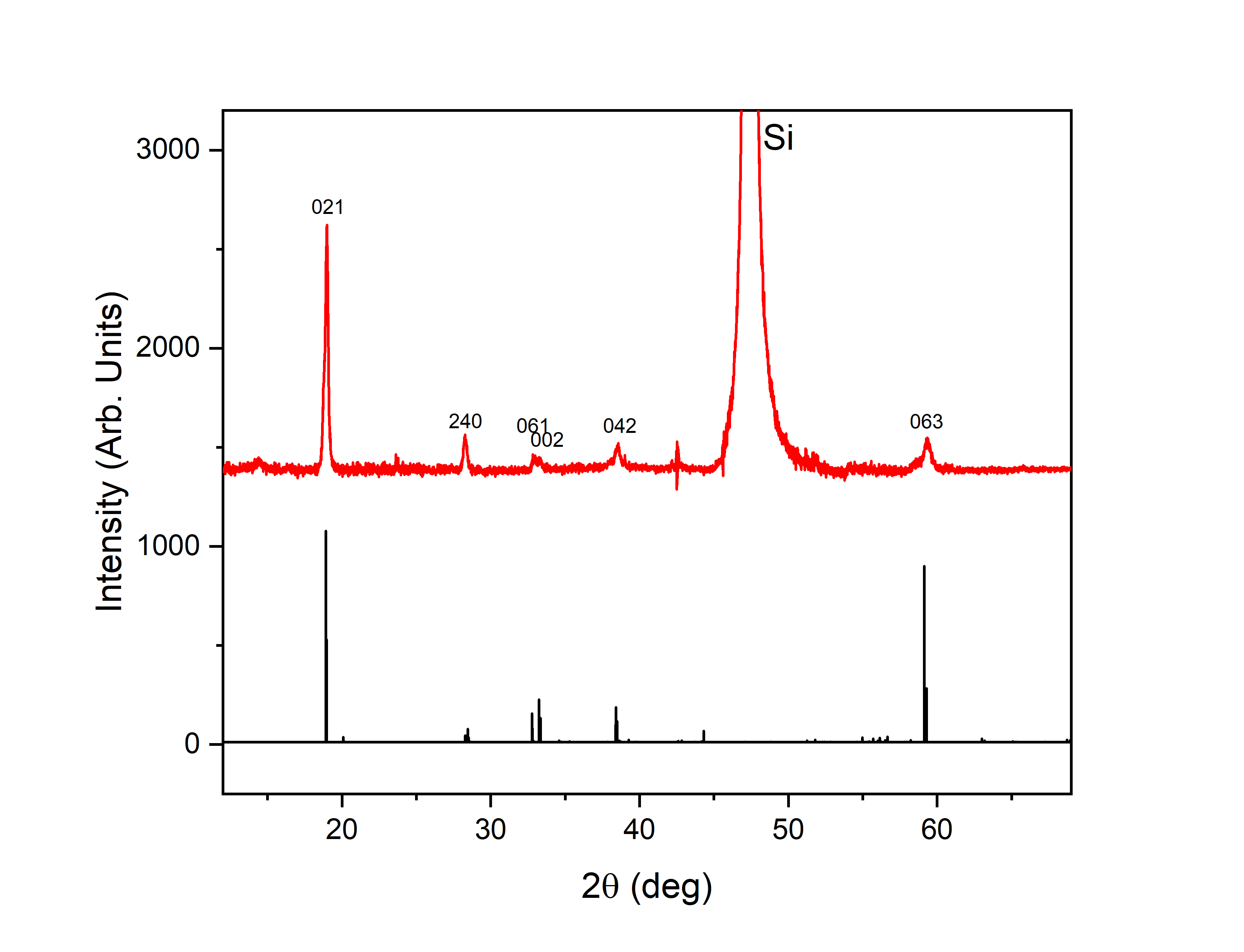


FIGURE S8: Raman spectra of the thin film and bulk samples.


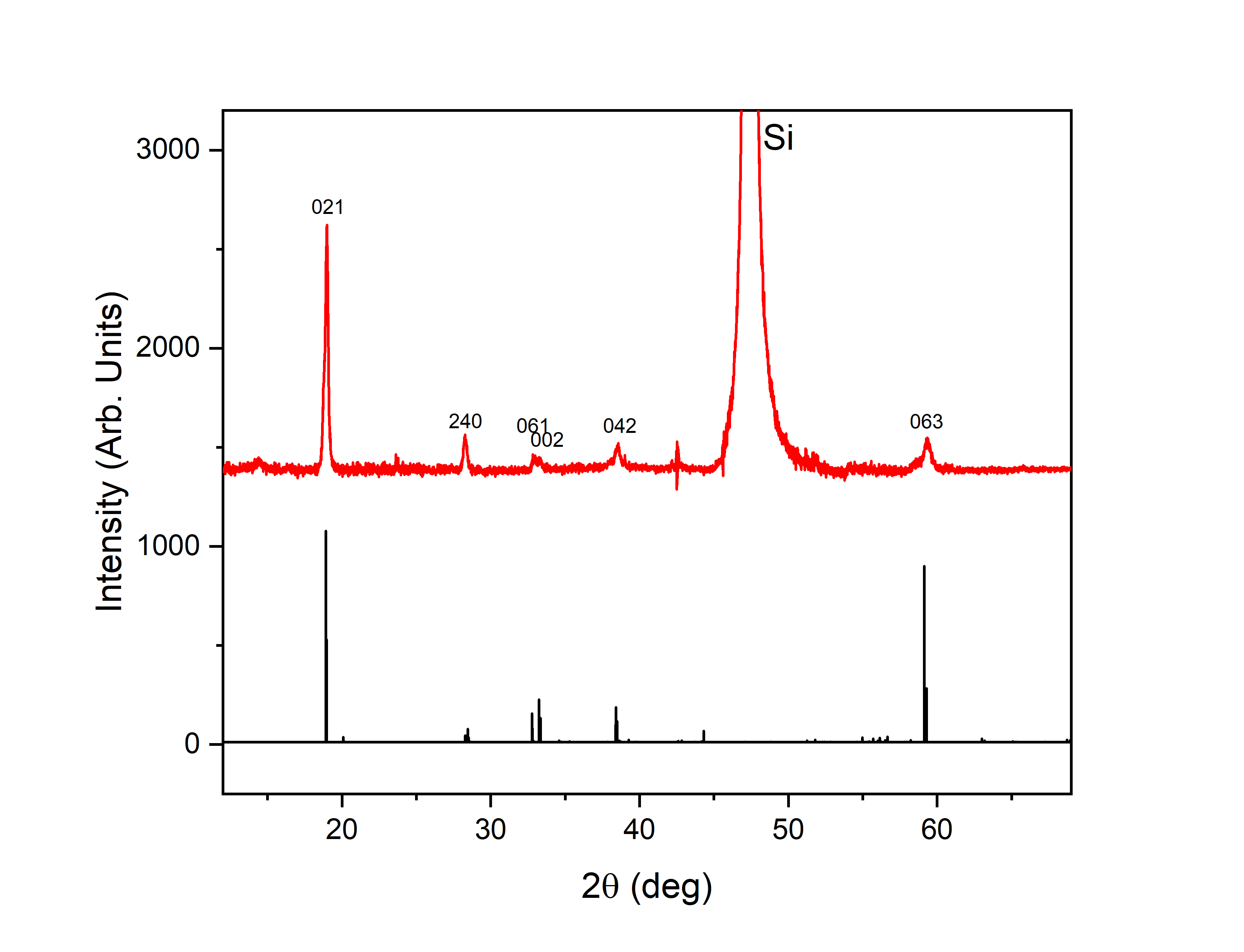

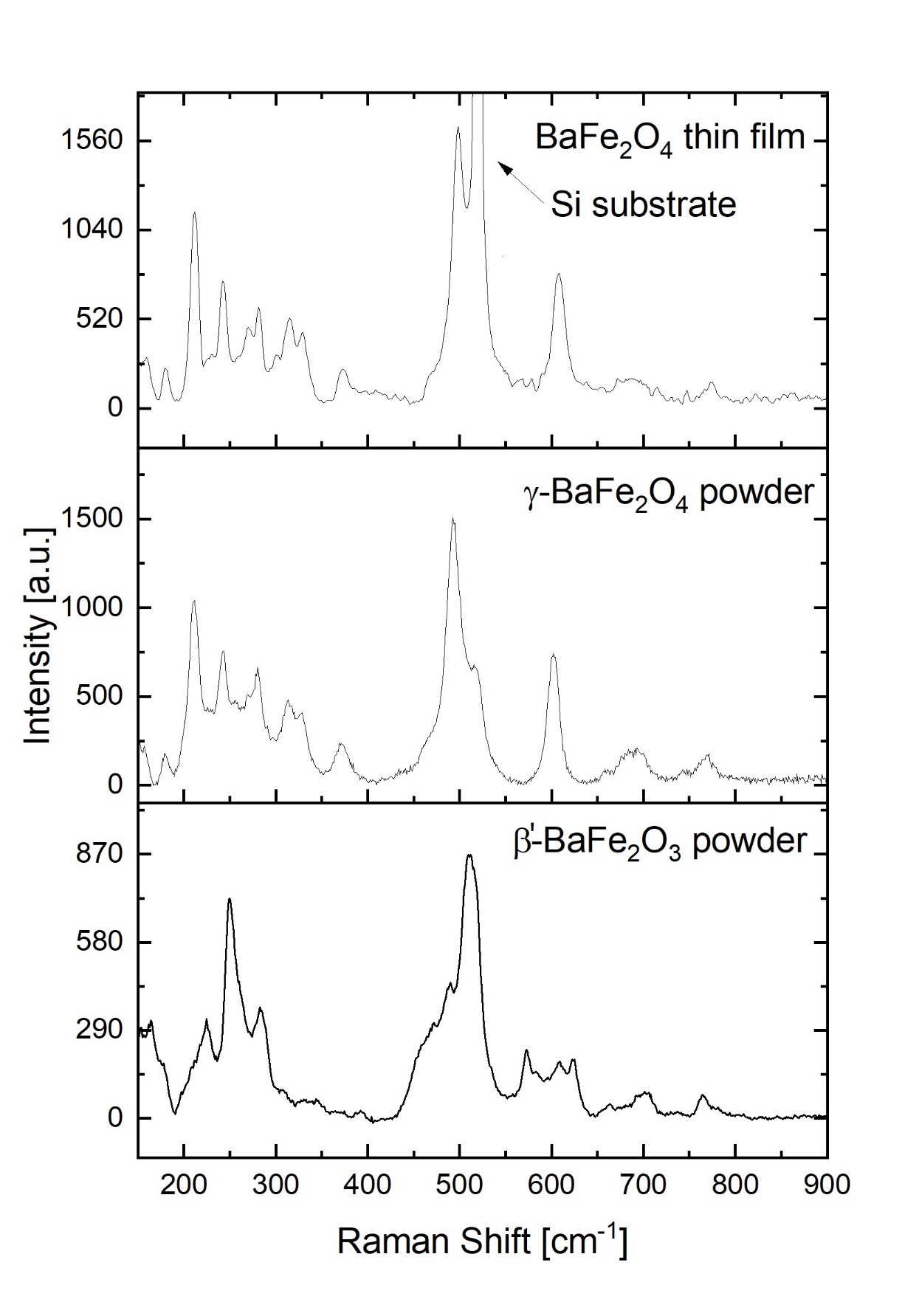


FIGURE S9: EDX spectrum of the thin film sample used for magnetic characterizations.


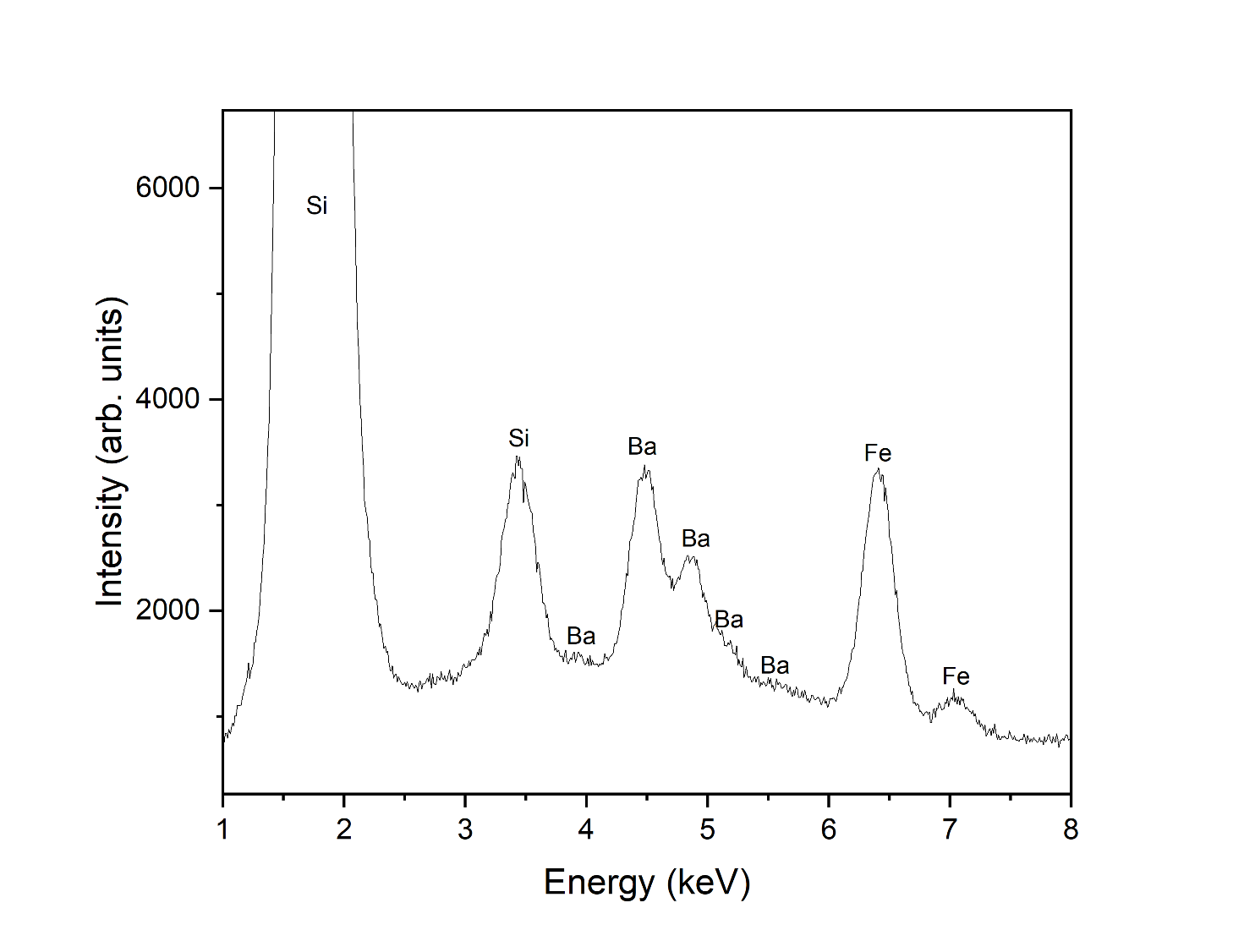


THIN FILM MAGNETIC CHARACTERIZATION PROTOCOL

A thin film of BFO grown by PED on Si/SiO_2_ substrate was characterized in terms of its M vs. H loop. The procedure used for such a study considered the strong disproportion of mass between the two components of the sample: the substrate, which represents almost the overall mass of the system, and the BFO thin film.

A protocol constituted by three steps was defined for this purpose:

(1) the substrate (constituted by Si covered with a thin layer of thermal SiO_2_) was measured cycling from 5T to –5T with a standard hysteresis loop SQuID protocol; (2) the same measurement protocol was performed on the same substrate after the growth via PED of the BFO film; (3) the raw data (emu/g) were point-by-point subtracted assuming *M_film_= M_blank+film_ – M_blank_*, and then plotted scaling the unit to μ_B_/F.U. in Fig. 2c of the manuscript, for consistency with the other magnetic measurements of the paper.

The measurements (1) and (2) are reported now in figure S10, while the subtraction is, as said, reported as Figure 2c.

FIGURE S10. a) M(H) loop measured between –5T and 5T for the pristine substrate of Si (black curve) and the system substrate + BFO thin film (blue curve); b) zoom of the substrate + BFO thin film loop between –2T and 2T.


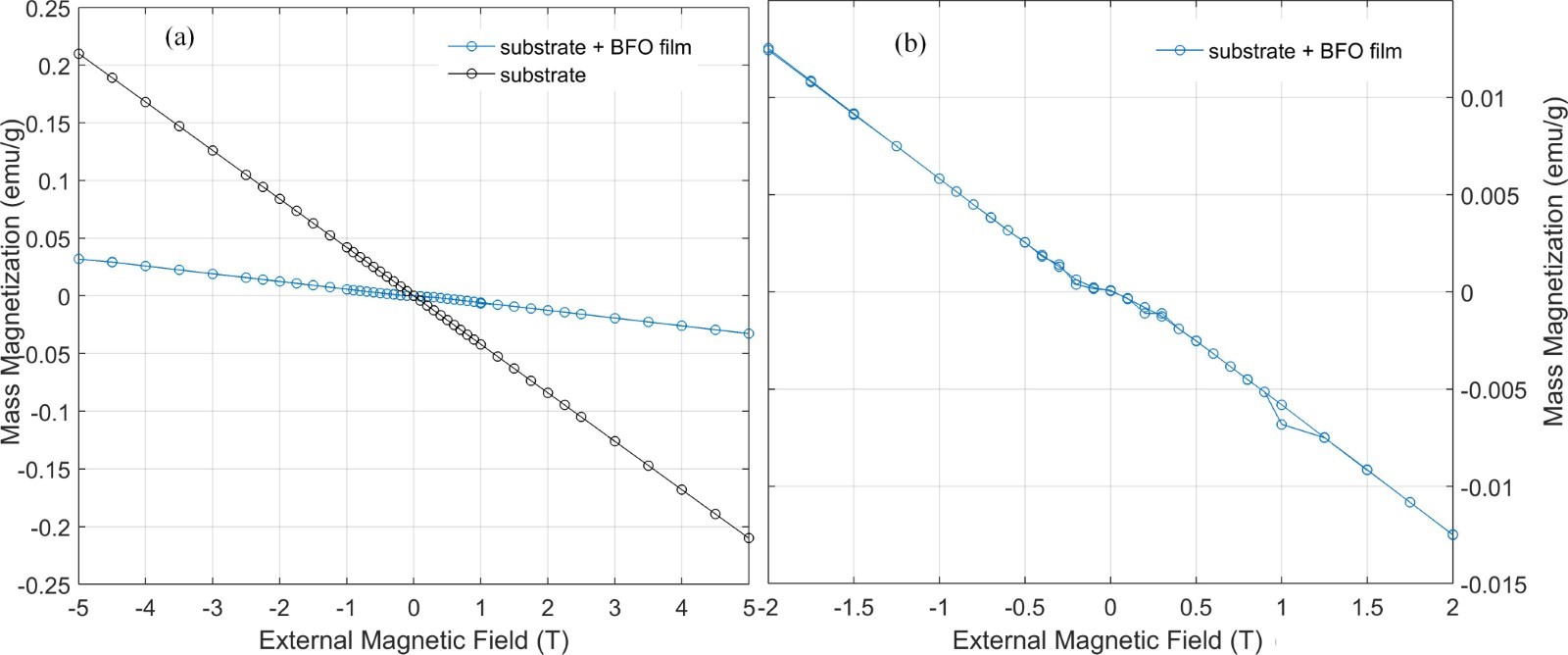


As it can be appreciated here, the substrate signal (black curve, Fig. S10a) is characterized by an almost ideal diamagnetic response, as expected for Si and an intense negative slope. In comparison, the sample constituted by substrate and thin film shows a diamagnetic susceptibility significantly reduced due to the presence of a superimposed positive trend of the magnetization coming from BFO (blue curve in Fig. S10a). Slight deviation from the linearity is solely observed in near zero-field conditions, where probably traces of ferromagnetic components, already observed in the bulk at higher concentrations, contribute to hugely flatten the negative slope of the diamagnetic blank (Fig. S10b).

X-RAY POWDER DIFFRACTION

FIGURE S11: Rietveld plot of PXRD data.

Refinement agreement factor: wR=7.05% for N_obs_=5741 and 59 variables.


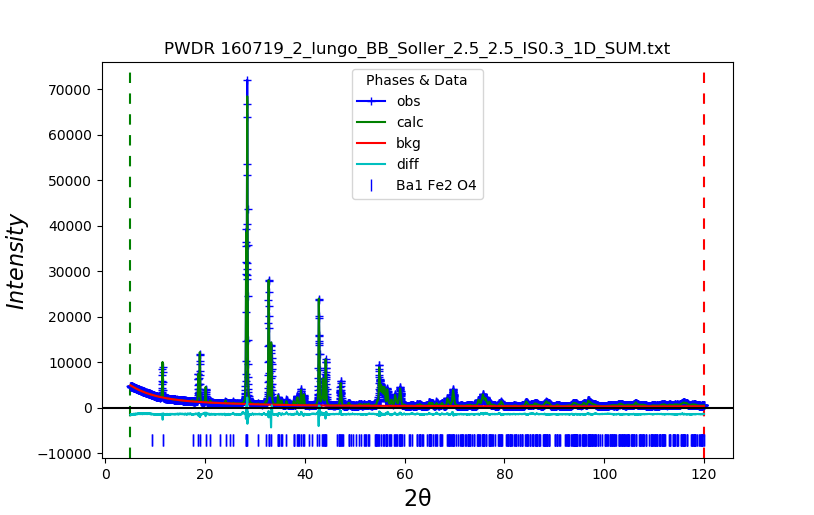


FERROELECTRICITY TEST PROTOCOL, DISPLACIVE CURRENTS AND VOLTAGE PROFILE VS. TIME AND DIELECTRIC CHARACTERISTICS OF THE SAMPLE IN HV REGIME

In Fig. S12 the utilized pulses sequence for the P(E) characterization reported in Fig. 2d of the main text is shown. The measurement was performed starting with a trapezoidal write pulse, characterized by rise-time and maximum amplitude retention time both of 5 ms, and read pulses of 100 Hz separated by delay time of 1 second. This specific protocol was necessary since the standard PUND determined the systematical dielectric breakdown of the sample.

FIGURE S12. “Opposite states” PN time sequence of triangular pulses applied for the P(E) measurement.


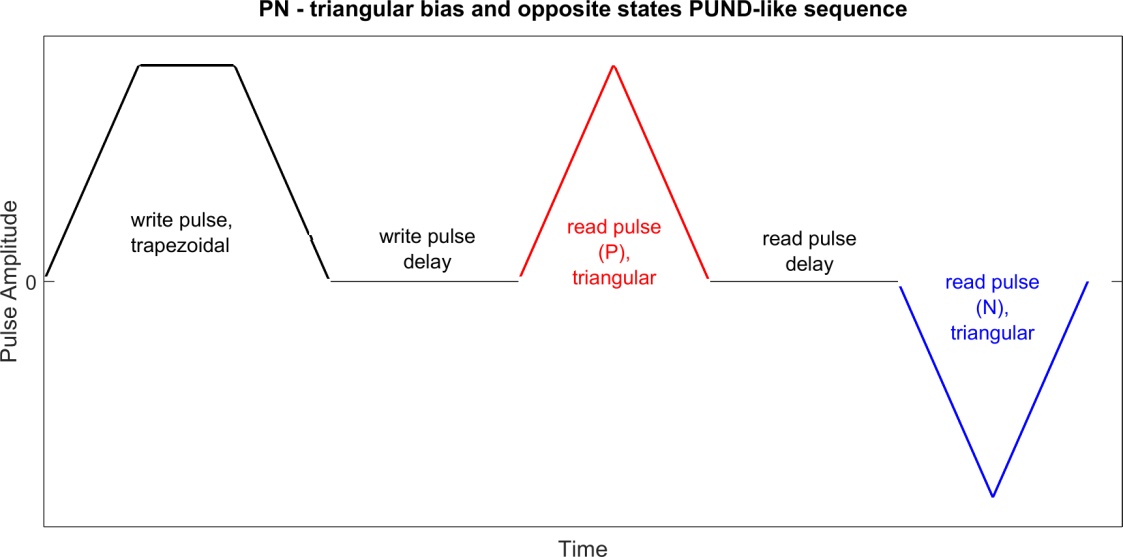


FIGURE S13. Measured displacive currents of a ceramic BaFe_2_O_4_ sample, and relative read pulses vs. time for the (left panel) P and (right panel) N branch of the triangular PN sequence.


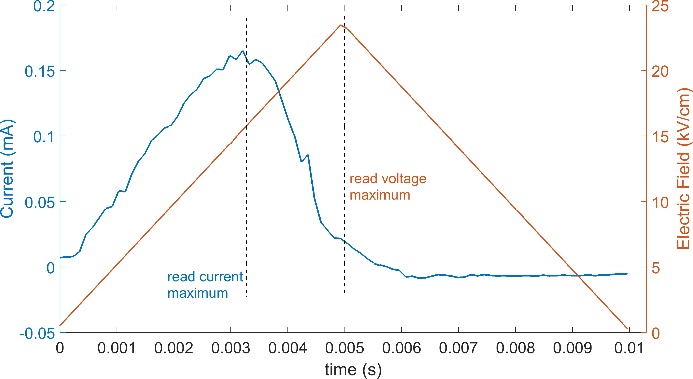

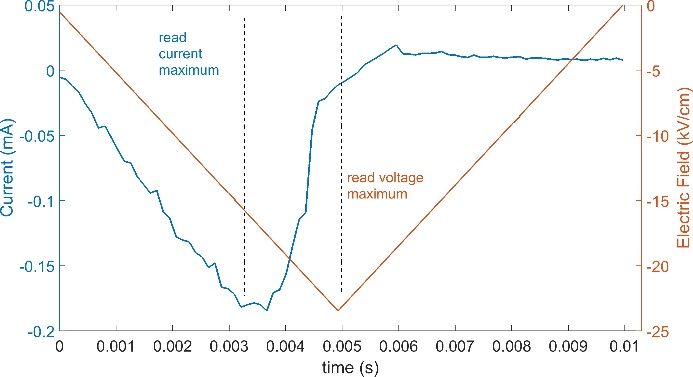


Figure S13 shows the switch to negative or positive value of the measured current occurring in the regime in which pulse derivative vs. time is negative or positive, respectively. This is consistent with the phenomenology expected for a dielectric material where I = dQ/dt = C dV/dt (C is the material capacitance), confirming that the sign of the current is concordant to the sign of voltage derivative.

FIGURE S14. Leaky dielectric character on BaFe_2_O_4_ emerging over ±20 kV/cm in the Tanδ plot vs Electric Field amplitude elaborated from a capacitance measurement performed in staircase mode with 100 Hz sinusoidal modulation.


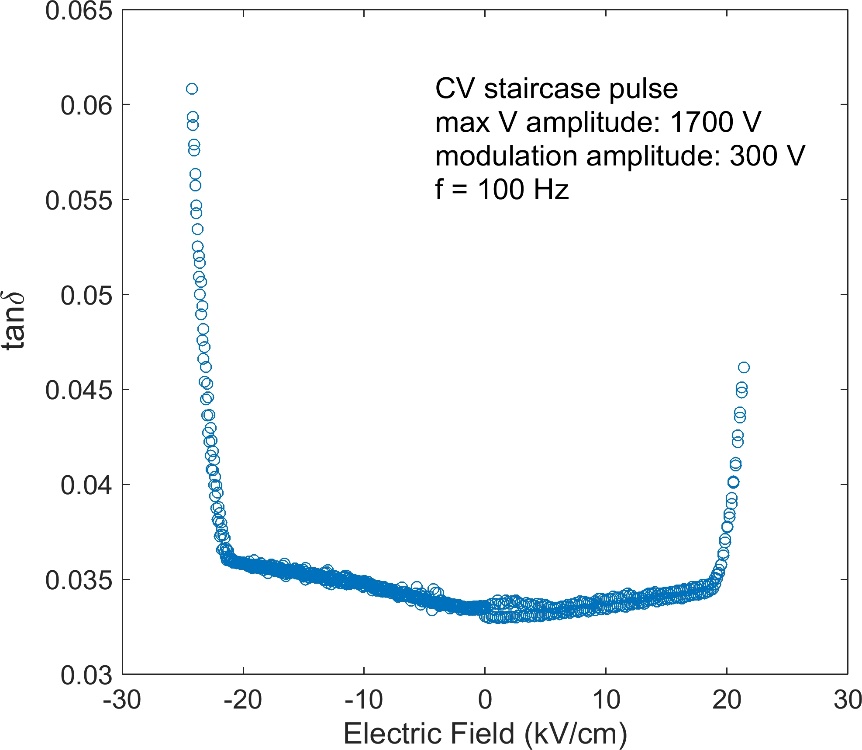


Measurable dielectric losses are evidenced by tanδ measurements, but not too high yet to define a significant deviation from a dielectric behavior. Tanδ precisely moves from around 0.035 to 0.06 at high field. Thus, the rotation of δ detected goes from 2° to 3.5° away from the ideal dielectric behavior (which is identified by δ= 0°). The opposite status, the ohmic/resistive behavior, is characterized by δ = 90°.

FIGURE S15: PFM images of a γ-BaFe_2_O_4_ single crystal. Vertical PFM amplitude (a) and phase (b). Lateral PFM amplitude (c) and phase (d).


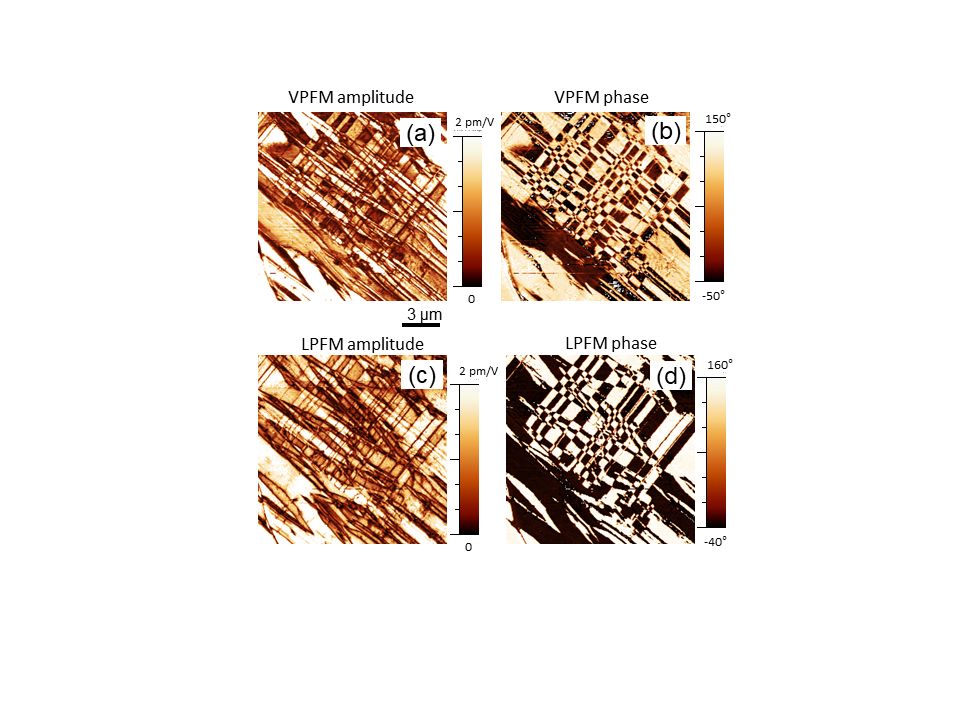


TABLE S4: Mode decomposition analysis [Perez-Mato, J. M., Orobengoa, D. & Aroyo, M. I. (2010). Acta Cryst. A66, 558-590.] of the DFT relaxed structure and of the single crystal x-ray model refined at RT. The mode decomposition is performed on the basis of the parent *P6/mmm* structure and shows good agreement when comparing DFT and experiments. The table reports the mode irreducible representation, the mode label, type and atomic site and the mode amplitude.

| Irreducible representation | Symmetry mode label and atomic site | Mode amplitude:  Relaxed structure | Mode amplitude: Experimental X-ray single crystal at RT |
| --- | --- | --- | --- |
| Γ_3_^-^ | dsp: O2 B3_u_(a) | 2.94462 | 2.94887 |
| R_1_ | occ: O1 A1’_1_(a) | 1.41421 | 1.41421 |
|  | dsp: Ba1 E1_u_(a) | 0.29892 | 0.23067 |
|  | dsp: Fe1 A2’’_1_(a) | 0.87520 | 0.88871 |
|  | dsp: Fe1 A2’’_2_(a) | 0.15097 | 0.13898 |
|  | dsp: O1 E’_1_(a) | -0.08917 | 0 |
|  | dsp: O1 E’_2_(a) | -0.06645 | 0 |
|  | dsp: O2 B1_u1_(a) | -0.52419 | -0.52653 |
|  | dsp: O2 B1_u2_(a) | -0.83849 | -0.68522 |
| Γ_6_^-^ | dsp: Ba1 E1_u_(a) | 0.0 | 0.0 |
|  | dsp: Fe1 E’(a) | -0.06067 | -0.03937 |
|  | dsp: O1 E’(a) | 0.79021 | 0.86759 |
|  | dsp: O2 B3_u_(a) | 0.30705 | 0.15063 |
|  | dsp: O2 B2_u_(a) | -0.20121 | -0.08022 |
| Γ_5_^+^ | dsp: Fe1 E’(a) | -0.02531 | -0.01649 |
|  | dsp: O1 E’(a) | -0.08908 | 0.07843 |
| M_2_^+^ | dsp: Fe1 E’(a) | -0.15785 | -0.13817 |
|  | dsp: O1 E’(a) | -0.86138 | -0.82591 |
|  | dsp: O2 B3_u_(a) | -0.04249 | -0.06407 |
|  | dsp: O2 B2_u_(a) | -0.08202 | -0.05481 |
| M_4_^-^ | dsp: Ba1 E1_u_(a) | 0.01910 | 0.01891 |
|  | dsp: Fe1 E’(a) | -0.09679 | -0.07567 |
|  | dsp: O1 E’(a) | -0.33383 | -0.36899 |
|  | dsp: O2 B2_u_(a) | -0.05370 | -0.04916 |
| R_2_ | dsp: Ba1 E1_u_(a) | 0.22471 | 0.18011 |
|  | dsp: O1 E’_1_(a) | 0.02003 | 0.0 |
|  | dsp: O1 E’_2_(a) | 0.01833 | 0.0 |
|  | dsp: O2 B1_u_(a) | 0.66953 | 0.64915 |

FIGURE S16: DFT spontaneous polarization of γ-BaFe_2_O_4_ calculated with the Berry phase polarization method (including both ionic and electronic contribution - left panel) or with a point charge model (i.e. by calculating the polarization “classically”, *P^PCM^ = Σ_cell_ Z_i_ |e|* ***u_i_****/Ω* where *e* is the electron charge, **u_i_** are the relative displacements of the ions, *Ω* is the unit cell volume and *Z_i_* are the nominal charge of each ion, i.e. +2, +3 and -2 for Ba, Fe, and O, respectively - right panel). The two approaches show quite a good agreement.


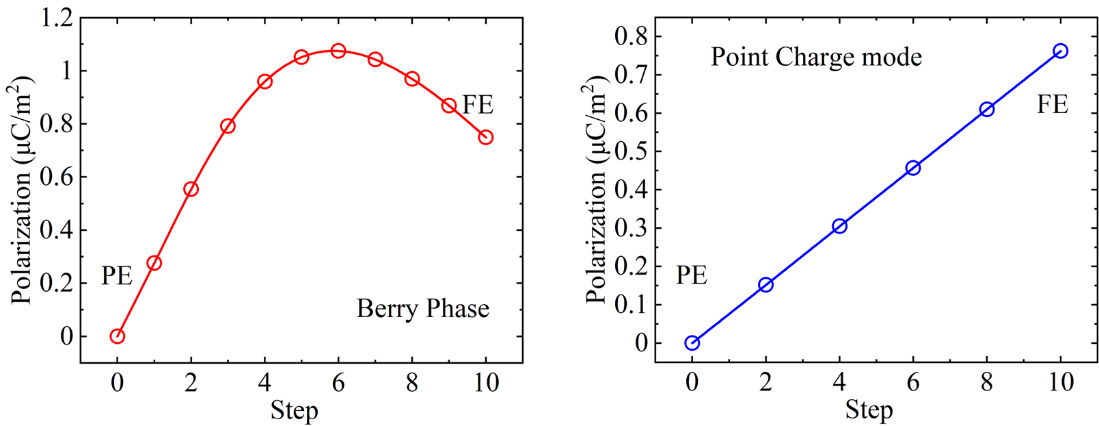


CONSTRUCTION OF A LIFSHITZ INVARIANT IN THE FREE ENERGY

In this section we will show that is possible to construct a Lifshitz invariant for BaFe_2_O_4_ indicating a possible instability towards a cycloidal state as it happens in BiFeO_3_. As it is described in the main text BaFe_2_O_4_ orders below T_N_ = 890 K with a propagation vector *k* = (1 0 0) which corresponds to the Y point of the first Brillouin zone. The experimentally observed G-type structure with moment aligned along the a-axis (G_x_) of the parent *Cmc2_1_* structure transforms as the mY_4_ irreducible representation (irreps). By considering that the G-type structure along z (G_z_) transforms as the mY_1_ irreps and the derivative operators $\frac{\partial}{\partial x}$ transform as Γ_4_ it is possible to construct the following antisymmetric invariant:

$G_{x}\frac{\partial G_{z}}{\partial x}-G_{z}\frac{\partial G_{x}}{\partial x}$ (I)

This invariant indicates an instability of the system toward the formation of a cycloidal structure with propagation vector along the *a* axis (Σ line of symmetry) of the parent *Cmc2_1_* structure.

FIGURE S17: Experimental PXRD patterns of γ-BaFe_2_O_4_ and of the metastable β’ polymorphs.


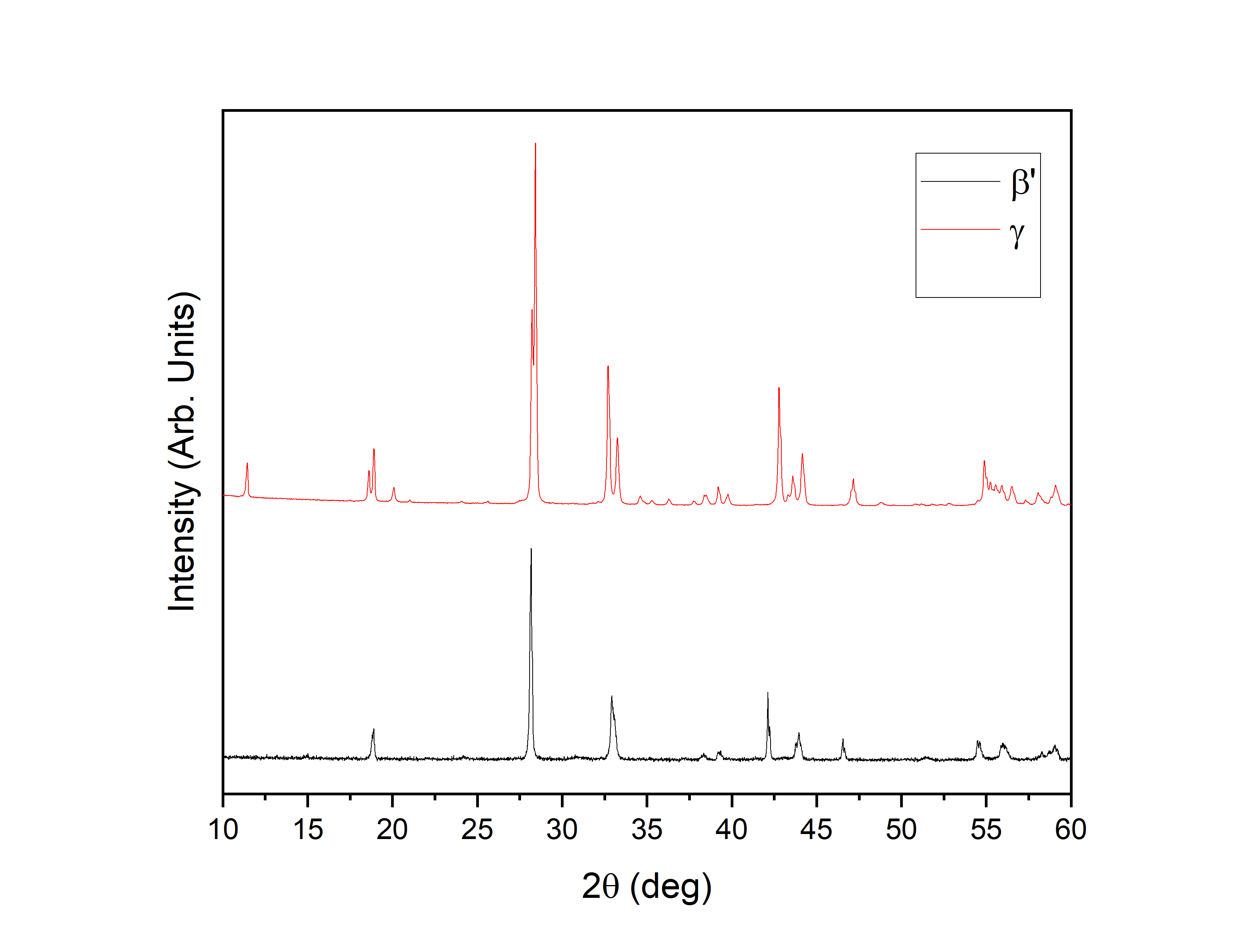

Supplement: Supplementary file 1 — Supplementery Information [file 41467_2022_35669_MOESM1_ESM.docx]
